# Fluorouracil (5-FU)-induced Cardiomyopathy

**DOI:** 10.7759/cureus.5162

**Published:** 2019-07-17

**Authors:** Nishanth Thalambedu, Yasir Khan

**Affiliations:** 1 Internal Medicine, Abington Hospital - Jefferson Health, Abington, USA

**Keywords:** 5-fluorouracil, cardiotoxicity, fluoropyrimidine, rectal cancer

## Abstract

Fluorouracil (5-FU) is a pyrimidine analog widely used in oncology. It is mainly used in the treatment of solid tumors. It is also used along with radiotherapy because of its radiosensitizing properties. As with all chemotherapy drugs, 5-FU is associated with adverse effects. Cardiotoxicity is one of them. It can present in a variety of ways and, sometimes, it can be lethal too. The reported case is of a 42-year-old male who presented with atypical chest pain after the first dose of 5-FU and was finally diagnosed with 5-FU-induced cardiotoxicity. His cardiac function normalized after the withdrawal of the drug.

## Introduction

Fluorouracil (5-FU) is a pyrimidine analog and has been in use for more than 40 years [1]. 5-FU is the second-most common drug associated with cardiotoxicity, after anthracyclines [2-3]. But the mechanism of damage and the optimal management of this condition is not well-defined. Our case presented with atypical chest pain, two days after the first dose of 5-FU. His EKG or electrocardiogram was within normal limits. He had mild troponin elevation, which prompted a further workup. Echocardiography showed reduced ejection fraction (EF) of 25%, with severe global hypokinesis. He underwent cardiac catheterization, which revealed normal coronaries leading to the suspicion of 5-FU-induced cardiotoxicity. He was discharged home after his acute symptoms resolved. His repeat echocardiogram (echo) after three months showed complete normalization of his cardiac function. He never received 5-FU after this episode. We reported this case to alert our fellow physicians about this rare adverse effect of a commonly used drug and current management options in the literature.

## Case presentation

A 42-year-old Caucasian male presented to the emergency department with non-specific complaints two days after his first round of chemotherapy with 5-FU. His symptoms started on the night of his chemotherapy session when he experienced multiple episodes of vomiting. On the following day, his vomiting continued to progress and was associated with a mild burning sensation in the chest, weakness, and fatigue. He did not complain of any shortness of breath, palpitations, or dizziness. His past medical history was remarkable for recently diagnosed T3N1 rectal cancer. The general physical exam was unremarkable except for mild generalized tenderness throughout the abdomen, with no signs of peritonitis.

**Figure 1:**
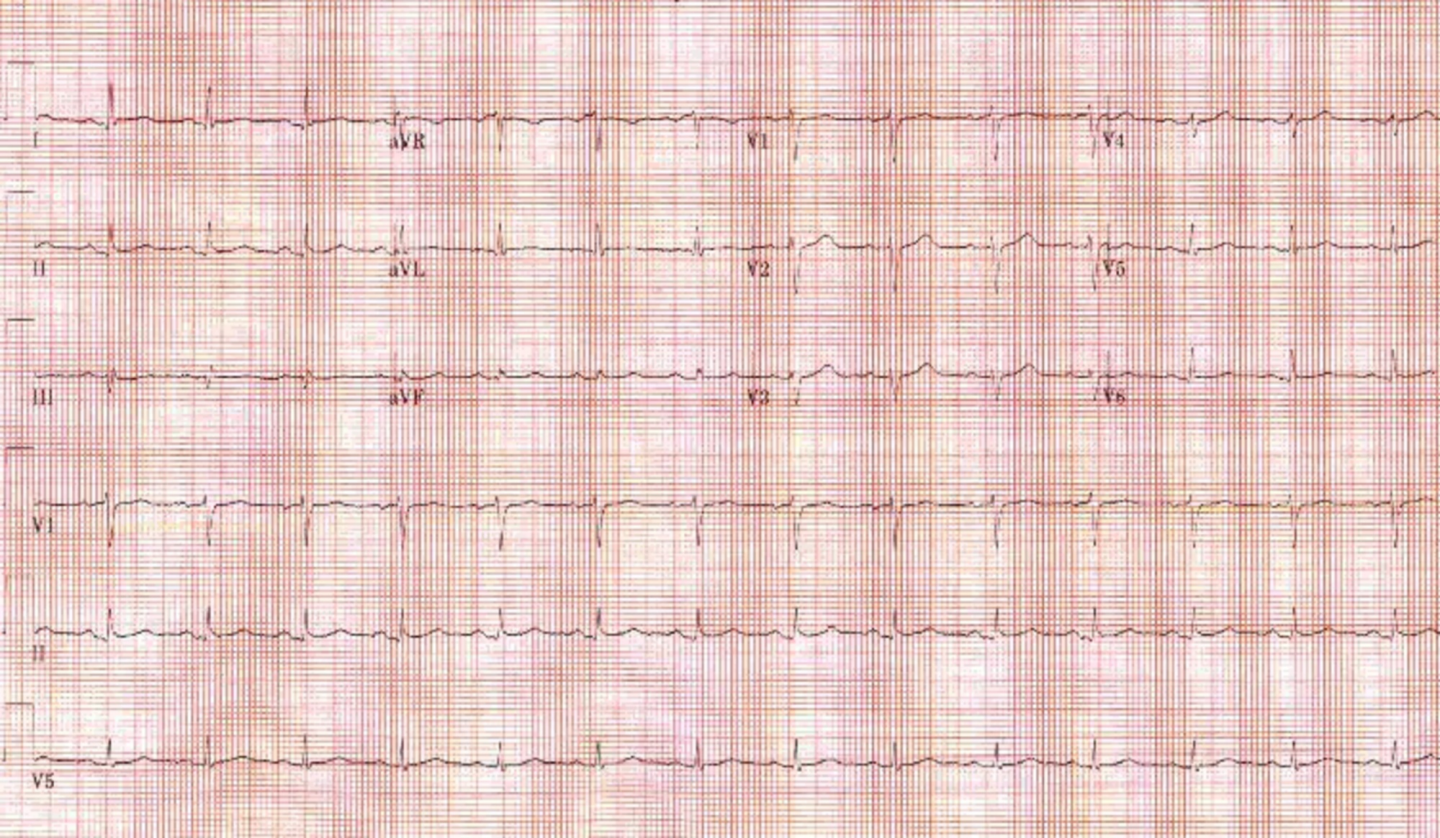
Electrocardiogram

Laboratory evaluation revealed a mild troponin elevation of 0.19 with normal EKG (Figure 1). The comprehensive metabolic panel and complete blood picture were normal.

Radiological investigations included an abdominal X-ray, which ruled out an obstruction. The patient also had echocardiography, which was remarkable for an estimated ejection fraction of 25% percent and severe global hypokinesis of the left ventricle. An initial mild troponin elevation in the setting of multiple episodes of vomiting and the absence of anginal symptoms was initially thought to be because of demand ischemia. But the further up-trending of troponin, along with remarkable echo findings, was concerning for acute cardiac process.

**Figure 2:**
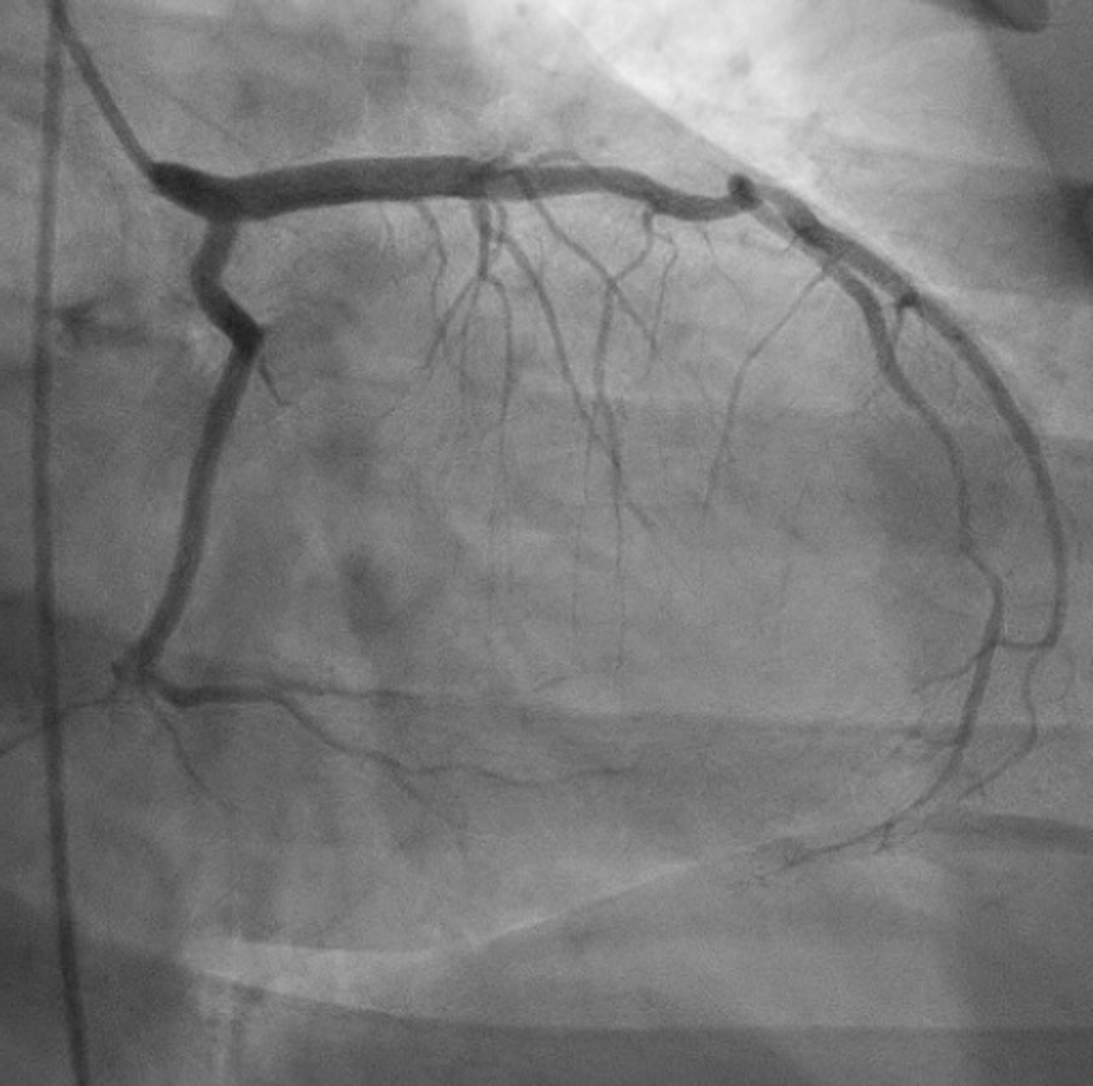
Left heart catheterization

**Figure 3:**
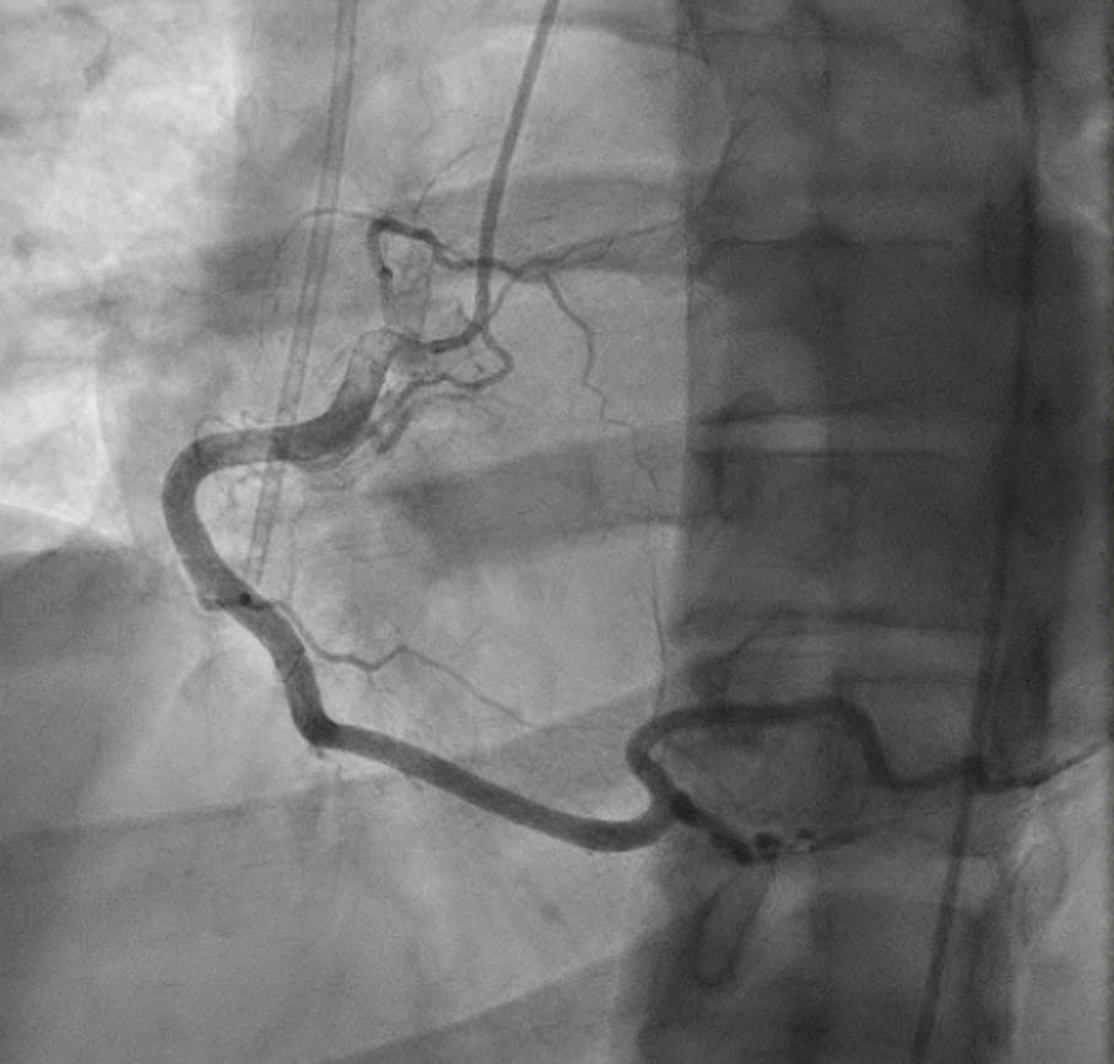
Left heart catheterization

He was treated with minimal intravenous isotonic fluids and antiemetics. He underwent cardiac catheterization, which revealed normal coronaries (Figures 2-3).

He was discharged home with beta-blockers and angiotensin-converting enzyme inhibitors (ACEi) after his acute symptoms resolved. He never received further chemotherapy. His repeat echocardiogram after three months showed complete normalization of cardiac function.

## Discussion

5-FU is a pyrimidine analog and the third-most commonly used chemotherapy drug for solid malignancies [4]. Cardiotoxicity has been reported as a rare adverse effect, with a reported incidence of 1.2% to 18% [5]. There are conflicting results from the literature regarding the risk factors in subjects who will have cardiotoxicity when they receive 5-FU. Patients with pre-existing cardiac disease and those without pre-existing disease but with risk factors for cardiac disease are prone to have 5-FU cardiotoxicity. Also, patients receiving infusional regimens are shown to have a higher risk than those receiving bolus regimens [6].

Multiple theories exist to explain 5-FU-induced cardiomyopathy. The more commonly accepted theories include coronary vasospasm, direct injury to the coronary endothelium and thrombus formation, and direct myocardial damage leading to global dysfunction [5]. 5-FU cardiotoxicity usually tends to occur during the first cycle of therapy, with a median time ranging between three and 18 hours [7-8]. 5-FU cardiotoxicity will manifest in a wide variety of fashions. It varies from atypical chest pain to sudden cardiac death [9]. But the most common presentation is found to be chest pain with or without rest [10].

In our case, the patient had a continuous infusion of 5-FU. He presented 48 hours after the infusion was started with atypical chest pain. His initial EKG showed no signs of ischemia. He did have a minimal troponin leak, which prompted us to have Echo, which showed severely reduced left ventricular function. He also underwent left heart catheterization, which showed clean coronaries.

**Figure 4:**
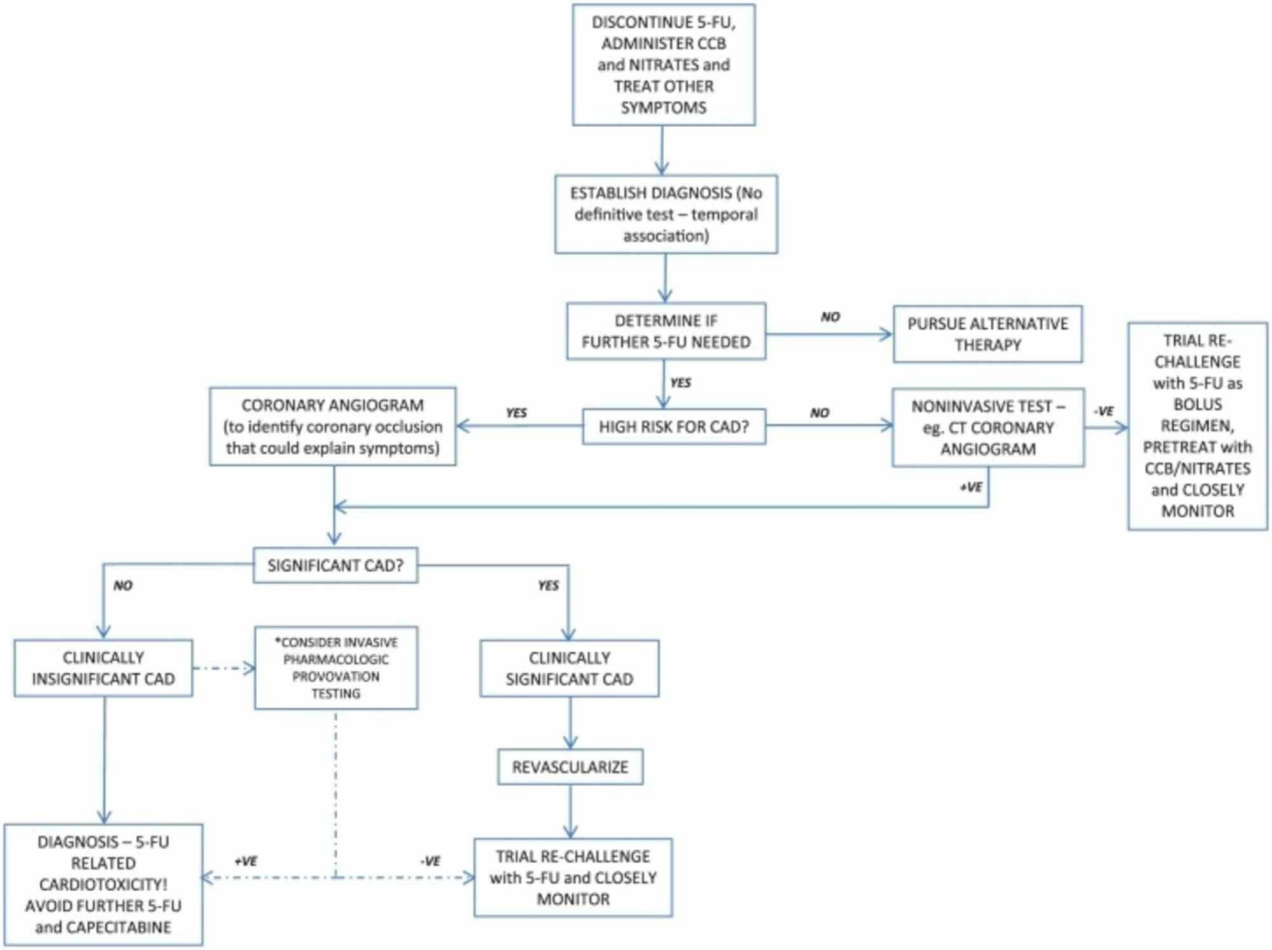
Primary management algorithm

The primary management (Figure 4) of 5-FU cardiotoxicity involves stopping the 5-FU infusion and treating the symptoms with anti-anginal agents, which showed a symptom-free state in 69% of subjects [11-13]. The next step is to establish a diagnosis. Non-invasive tests like EKG, Echo, cardiac troponins, and brain natriuretic peptide (BNP) will guide us towards the next steps. The decision to do an invasive test like a coronary angiogram will depend on the results of the non-invasive tests. Moving forward, the best treatment is to use non-fluoropyrimidine chemotherapy. If rechallenging with 5-FU is decided, it's recommended to use the bolus regimen instead of an infusional regimen [8].

Multiple preventive strategies have been studied. In a study by Eskilsson et al., verapamil 120 mg three times a day was given as a prophylaxis to the patients receiving a continuous 5-FU infusion, but it did not reduce the incidence of ischemia [14]. In a different study by Salepci et al., angiotensin II levels were measured in the patient group receiving bolus 5-FU infusions and noted no difference from the control group, thereby recommended prophylactic angiotensin-converting enzyme inhibitors (ACEi) should not be used to prevent cardiotoxicity [15]. More studies need to be done in this area to recommend guidelines.

In our patient, the initial troponin leak and echocardiogram findings of severe left ventricular dysfunction prompted us to take the patient for an angiogram, which showed clean coronaries. We discharged the patient on beta-blockers. The patient pursued alternative therapy and never received 5-FU again. In three months, his cardiac function normalized.

## Conclusions

5-FU cardiotoxicity is a rare adverse event but clinicians should be aware of this complication because of the morbidity and mortality associated with it. The mechanism of cardiotoxicity is poorly understood. Risk is relatively higher in those receiving continuous infusions rather than the bolus regimen. The management of this complication requires immediate stopping of the drug followed by establishing a diagnosis. Rechallenge is not routinely recommended. Till date, no proven preventive strategies exist.
